# Tobacco industry attempts to frame smoking as a 'disability' under the 1990 Americans with Disabilities Act

**DOI:** 10.1371/journal.pone.0188188

**Published:** 2017-11-27

**Authors:** Yvette van der Eijk, Stanton A. Glantz

**Affiliations:** 1 Center for Tobacco Control Research and Education, University of California San Francisco, San Francisco, California, United States of America; 2 Philip R. Lee Institute for Health Policy Studies and Department of Medicine, University of California San Francisco, San Francisco, California, United States of America; Iowa State University, UNITED STATES

## Abstract

Using the Truth Tobacco Industry Documents Library and Congressional records, we examined the tobacco industry’s involvement with the 1990 Americans with Disabilities Act (ADA). During legislative drafting of the ADA (1989–1990), the Tobacco Institute, the tobacco industry’s lobbying and public relations arm at the time, worked with industry lawyers and civil rights groups to include smoking in the ADA’s definition of “disability.” Focus was on smoking as a perceived rather than actual disability so that tobacco companies could maintain that smoking is not addictive. Language that would have explicitly excluded smoking from ADA coverage was weakened or omitted. Tobacco Institute lawyers did not think the argument that smokers are “disabled” would convince the courts, so in the two years after the ADA was signed into law, the Tobacco Institute paid a lawyer to conduct media tours, seminars, and write articles to convince employers that hiring only non-smokers would violate the ADA. The ultimate goal of these activities was to deter employers from promoting a healthy, tobacco-free workforce and, more broadly, to promote the social acceptability of smoking. Employers and policy makers need to be aware that tobacco use is not protected by the ADA and should not be misled by tobacco industry efforts to insinuate otherwise.

## Introduction

The 1990 Americans with Disabilities Act (ADA) is a comprehensive civil rights law that prohibits discrimination against people with disabilities and provides equal opportunities to participate in all aspects of life [1]. It became law on July 26, 1990, after two years of legislative drafting and a decade of lobbying by civil rights organizations.

The association between the ADA and smoking–specifically using the ADA as a tool to protect smokers from differential hiring policies–is not an association many would instinctively make, since the tobacco industry has argued smoking is a lifestyle choice, not a disability. However, the tobacco industry has a long history of working to maintain a legal and policy environment that supports use of its products and makes it difficult to implement tobacco control policies, even when the connection of the policies in question to tobacco use is not obvious. Notable examples include the shaping of regulatory reforms, known as “Better Regulation” to make implementing regulations more difficult in the European Union [2], and the state-level “smoker’s rights” laws in the USA, which were framed as civil rights legislation to protect legal off-the-job activities [3]. Tactics are often subtle, with tobacco industry involvement hidden. The industry also works to shape perceptions of laws that support the industry’s position even when the language in the law does not, as it does when promoting the perception that legitimate public health promotion is “illegal lobbying” [4].

While the ADA was being developed, evidence was mounting that smoking and exposure to secondhand smoke was detrimental to health and work productivity. Health insurance companies increased premiums for smokers [5], and some employers started charging smokers more for health insurance, creating smokefree workplaces, and hiring only nonsmokers. By 1985, 15% of U.S. companies allowed or mandated a preference for nonsmoking job applicants [6]. Tobacco companies responded by forming alliances with organized labor [7], exemplified by the Tobacco Institute’s Labor Management Committee [8], to oppose smokefree workplace rules. In 1989–1993, tobacco companies also funded the American Civil Liberties Union (ACLU), a powerful civil rights group [9], to help the companies push for “smoker’s rights” laws that prohibit employers from only hiring nonsmokers, arguing that this represented “lifestyle discrimination” [3]. Many of the laws had loopholes however, allowing exceptions for job applicants, certain professions, or cases where there was a rational basis for discrimination [10].

Some lawyers also argued that hiring only non-smokers was “disability discrimination” against smokers. In 1988, a job applicant who smoked successfully sued an employer under Minnesota’s handicap law, for refusing to hire smokers because they are at a higher risk of developing disabilities later in life [11,12]. After the ADA passed, some legal scholars argued that smoking is a “disability” on the grounds that smoking is an addiction that interferes with normal everyday functions, including working [13,14]. Subsequent legal analyses and court cases rejected this argument [3,6,15]. The text of the ADA is, however, unclear on whether smokers are covered by the ADA [5,6]. We examine the tobacco industry’s involvement in the ADA, specifically: (1) legislative drafting of the ADA on possible inclusion of smokers in the ADA’s definition of disability, and (2) attempts to convince employers that smokers are protected by the ADA.

## Methods

We searched internal tobacco industry documents in the Truth Tobacco Industry Documents Library (https://www.industrydocumentslibrary.ucsf.edu/tobacco/), a publicly accessible, online library of previously secret tobacco industry documents mostly released through litigation against tobacco companies. The first author conducted the search from September to November 2016 using a snowball search, the standard method for tobacco industry documents research [16,17]. We started by doing a keyword search with the term “Americans with Disabilities Act”. After identifying relevant documents we examined adjacent documents in the collection (by Bates numbers, sequential numbers stamped on every page of documents as they were being produced in litigation). We also conducted follow-up searches in the documents library by doing a keyword search of names of individuals (e.g. “John Fox”), organizations (e.g. “Dole Foundation”) and lawsuits (e.g. Daniels v DCA) mentioned in relevant documents. This process was repeated until we reached saturation, i.e., until no new relevant documents were identified. We also searched Congressional records based on key dates and events mentioned in the documents, including public hearings, committee reports, and votes. The Congressional records were then used to inform further searches in the Truth Tobacco Industry Documents Library.

We retrieved approximately 5,300 documents. We filtered out irrelevant documents, such as news articles and legal contracts that mention the ADA but provide no information on the tobacco industry’s ADA strategy. Many documents were duplicated, for example as a result of the same report being sent to multiple recipients (each which creates a duplicate copy of the report in the Library). These duplicates were also filtered out. We used 54 documents for this analysis. This analysis pertains to the ADA as originally passed in 1990, not the subsequent amendments enacted in 2008.

## Results

### The Tobacco Institute’s efforts to change ADA language

#### Attempts to reword ADA “definitions”, November–December 1989

As of November 14, 1989, there were two parallel versions of the ADA bill (Table 1). The Senate version (S.933) excluded all current psychoactive substance use disorders, and therefore tobacco use, from ADA coverage [18]. This exclusion was the result of the “Armstrong Amendment,” a revision of ADA section 511, “definitions” [19], by Senator William Armstrong (R-CO). The House version (H.R.2273) contained language in “definitions” similar to the Armstrong Amendment, but with a subtle rewording that only excluded substance use disorders resulting from current use of illegal drugs, so did not exclude tobacco use from ADA coverage.

**Table 1:** Key events in the ADA legislative drafting process, May 1989 –July 1990.

Date	Event (House)	Event (Senate)
1989, May 9	ADA companion bill is introduced in the House as H.R.2273 [20].	ADA bill is introduced in the Senate as S.933 [19].
1989, Sep 7		Senate debates and passes S.933, and adds the Armstrong amendment which excludes all psychoactive substance use from the definition of “disability” [18,19].
1989, Nov 14	Mark-up of H.R.2273 by House Education and Labor Committee [20], which votes in favor of a modified H.R.2273 which excludes illegal drug use and alcohol abuse, but not nicotine use from the definition of “disability” [21].	
1989, Nov 28	Letter in the Tobacco Institute records (involving law firm Wunder, Ryan, Cannon & Thelen) describes plans to weaken the Armstrong amendment, by diluting its language and moving it to section 103 [22].	
1990, Dec 5 (estimate)	Letter in the Tobacco Institute records (involving law firm Wunder, Ryan, Cannon & Thelen) describes plans to weaken the Armstrong amendment, by adopting language to include voluntarily acquired conditions such as obesity [21].	
1990, Mar 3	Mark-up of H.R.2273 by House Energy and Commerce Committee [20].	
1990, Mar 11	Memo from Michael Forscey (lawyer at Wunder, Ryan, Cannon & Thelen) to Jeff Schlagenhauf (assistant to Congressman Thomas Bliley Jr, R-VA) describes plans to neutralize Congressman Bob Whittaker’s (R-KS) proposed language for the “construction” section by negotiation between Whittaker and Bliley [23].	
1990, May 15	House report by four House Committees on H.R.2273 recommends passage.[20]	
1990, May 17	House debates H.R.2273.[20]	
1990, May 22	House passes H.R.2273.[20]	Senate passes S.933.[19]
1990, Jul 12	Conference report on S.933 is passed by House.[19]	
1990, Jul 13		Conference report on S.933 passed by Senate.[19]
1990, Jul 26	S.933 signed into law by President George H.W. Bush.[19]	

A plan sent to the Tobacco Institute on November 28, 1989 describes how Institute lawyers had weakened the Armstrong Amendment [21,22]. The plan lists no author(s), although an attachment was prepared by the law firm Wunder, Ryan, Cannon & Thelen which worked for the Tobacco Institute on ADA projects [24,25]. Michael Forscey, a lawyer there, had worked for the Tobacco Institute since at least 1987 [26], was a key member of the Tobacco Institute’s Labor Management Committee, and served as legal counsel to the Senate Labor Committee before he worked with the Tobacco Institute [27].

The Tobacco Institute considered the ADA as a potential “legal recourse against employers who refuse to hire smokers” [21]. The Armstrong Amendment was thereby a threat, while the House version was a successful product of their collective efforts:

The House Education and Labor Committee recently reported another version of the ADA which favorably changes the language of the Armstrong amendment, due in large part to our cooperative efforts with civil rights groups. The House version of the bill now excludes from the ADA’s protection only illegal psychoactive substance use disorders. Thus, the change from the Senate version to the House version adds alcohol, caffeine and nicotine use to the conditions which clearly fall under the ADA [21, pg. 1].

The plan suggested including voluntarily acquired conditions such as obesity in ADA coverage. Coverage could be established by analogy if smoking, like obesity, was considered a voluntary behavior that could be remedied through lifestyle change. The plan also suggested that, by analogy to conditions such as HIV infection, smoking could be considered a perceived rather than actual disability, which would not require admitting that smokers were “addicted,” only that others perceived them as being addicted.

To ensure smokers would not be excluded from ADA coverage, the Tobacco Institute planned to dilute rather than eliminate the Armstrong Amendment using a three-part plan [22]. First, the reference to “psychoactive substance use disorders” would be eliminated from the Armstrong Amendment by arguing that this was redundant to other provisions of the bill. The reasoning was that illegal substance use was already excluded, and where substance use was legal, “job performance should be the only permissible employment criteria” [22, pg. 29]. Second, the Armstrong Amendment would be moved from section 511 (“definitions”) to section 103 (“defenses”). This would put a higher burden of proof on employers, who would have to prove the presence of a listed condition (e.g. alcoholism) before denying a discrimination claim, as opposed to requiring an employee to prove the absence of a listed condition. Third, a provision would be added to require employers to “establish the job-relatedness of the condition before that condition may serve as the basis for an employment decision” [22, pg.29].

#### Plans to reword ADA “construction,” March 1990

As of November 1989, both S.933 and H.R.2273 included an anti-preemption provision in section 501(b), “construction,” to protect state and local disability rights laws that gave greater protection than the ADA [28]. In March, 1990, the House Energy and Commerce Committee marked-up H.R.2273 [29]. Meanwhile, tobacco control advocates proposed to reword “construction” so that the ADA could not preempt smoking restrictions. Congressman Bob Whittaker (R-KS) of the House Energy and Commerce Committee proposed inserting the following language into “construction:”

Nothing in this act shall be construed to prohibit the prohibition of smoking in places of public accommodation, transportation or places of employment.[23, pg.4]

Whittaker also proposed adding the following sentence in the committee report explaining “construction”:

A person who uses a tobacco product and is addicted to nicotine would not be considered disabled under this act solely on the basis of their use of tobacco or addiction to nicotine [23, pg.4].

Whittaker’s report language would nullify any argument that smoking was a disability under the ADA, even if attempts to weaken the Armstrong Amendment were successful. Forscey thus described Whittaker’s report language as “a big hurdle” [23, pg.1]. Forscey presented a plan in which a substitute language, to be offered by Congressman Thomas Bliley, Jr (R-VA) would replace Whittaker’s language. Bliley, also a member of the House Energy and Commerce Committee [30], was a strong advocate for the tobacco industry [9]. Bliley’s proposed substitute language still protected state and local smoking restrictions from preemption by the ADA, but unlike Whittaker’s language did not exclude smokers from ADA coverage. The plan was for Bliley to negotiate compromise language with Whittaker. Failing that, if Whittaker offered an amendment in committee, it would be defeated and replaced with Bliley’s language. John Dingell (D-MI), chair of the House Energy and Commerce Committee, would be asked to help defeat it [23].

#### Outcome of Tobacco Institute attempts to change ADA language, May–July 1990

As the ADA bill moved through the legislative process (Table 1), the language in section 511, “definitions”, remained consistent with the House version, which the Tobacco Institute considered to be more favorable [31]. In the final law, section 511 only excludes “psychoactive substance use disorders resulting from current use of illegal drugs” from ADA coverage [4]. There is no mention of including voluntarily acquired conditions such as obesity. The Tobacco Institute appears to have been successful in weakening the language of the Armstrong Amendment, but not in moving it to section 103 or explicitly including voluntarily acquired conditions in the ADA definition of disability. In the final ADA law and report, section 501(b), “construction”, is very similar to Bliley’s proposed language [32]. There are no records of Whittaker proposing an amendment for this section in committee [33], which indicates that the Tobacco Institute successfully weakened Whittaker’s proposed language by negotiation with Bliley before May 15, 1990.

The ADA as signed into law on July 26, 1990 was ambiguous on whether smokers were considered disabled under the ADA, or whether employer policies against hiring smokers would violate the ADA.[32] Specifically, to be protected by the ADA, one must have a disability, defined broadly as:

(A) a physical or mental impairment that substantially limits one or more of the major life activities…;

(B) a record of such an impairment; or

(C) being regarded as having such an impairment [4, Sec. 12102].

The ADA does not list all the covered impairments although certain conditions, notably illegal substance use disorders, are specifically excluded. Under prong C, the “perception test,” one need not have a physical or mental impairment to be considered disabled [1]. For example, the perception test might apply to people with asymptomatic HIV who are not mentally or physically impaired, but treated as such because of the stigma surrounding their condition. Prospective and current employees with disabilities are protected under Title I.

### The Tobacco Institute’s ADA public relations program

#### Plans for a 1991 ADA public relations program

In February 1990, the Tobacco Institute started putting together an ADA public relations program for 1991 to deter employers from hiring only nonsmokers on the grounds that doing so would violate the ADA. John Fox, a partner at Pettit & Martin law firm, was the main lawyer on this program. Fox had consulted for the Tobacco Institute since 1985, writing legal briefs and participating in media tours to deter smokefree workplace rules [34]. Fox worked with staff in the Tobacco Institute’s Public Affairs Division: Martha Rinker, Karen Fernicola, Susan Stuntz and Martin Gleason [35]. Initial ideas for the program are described in a Tobacco Institute memo, five months before the ADA was signed into law. At that point, Forscey had reported to the Tobacco Institute that the ADA might protect smokers as “disabled persons” [36]. Stuntz wrote to Gleason that, since the ADA would prevent hiring bans against smokers, a public affairs program should be developed by “using the Fox legal briefings and any other avenues the public smoking folks deem appropriate” [36].

The Tobacco Institute Public Affairs Division’s “1991 Proposed Budget and Operating Plan” described its ADA program as “a public relations program surrounding enactment of the Americans with Disabilities Act, focusing on its implications for employers attempting smoker hiring bans” [37, pg. 2]. The program would “educate” employers on the implications of the ADA via workplace legal briefings, law journal articles and other materials targeted to employers. The Tobacco Institute planned to “continue to produce and promote” Fox’s seminars on the ADA, and include references to smoking in these seminars [37, pg. 172]. By then, Fox was already conducting ADA briefings through Pettit & Martin and the National Employment Law Institute (NELI), an education organization with Fox on its board of directors [38,39]. In November 1990, Fox told the Tobacco Institute how he subtly framed smoking as a disability in his NELI briefings:

…three of my five… briefing tours allowed the “smokers as disabled” issue to arise naturally from the context of the presentation without the need for me to raise it. Rather, I was in the enviable position to simply respond to questions about whether smokers [are] disabled… [40, pg. 1].

Meanwhile, Tobacco Institute lawyers Forscey and John Rupp advised the Institute that the argument that smokers were “disabled” under the ADA would unlikely be accepted by the courts, but could be used to “persuade corporate decision makers” to not exclude smokers from the workforce [41]. Rupp, a lawyer at Covington & Burling, had worked for the Tobacco Institute since 1981, managing its scientific and regulatory responses to issues related to secondhand smoke [42]. A restricted memo dated December 5, 1990, from Forscey to Rupp, suggests that tobacco companies had sought legal advice on whether the ADA could be used as a basis for litigation against employers which hired only nonsmokers. No tobacco industry documents were found indicating tobacco industry involvement in such litigation. The Tobacco Institute’s ADA strategy in 1991 was focused on persuading employers rather than litigating in courts, since the argument that smokers are disabled under the ADA was expected to have little or no legal merit.

#### Outcome of the Tobacco Institute’s ADA public relations program, 1991

Throughout 1991, Fox produced several employer training materials and publications for the Tobacco Institute (Table 2). These materials all framed smoking as a perceived rather than actual disability, and references to smoking were not made directly but by analogy to other lifestyle-related conditions such as obesity. This approach subtly reinforced the impression that smoking was a disability under the ADA, and that discriminating on the basis of smoking status violated the ADA. This made it easier for the Tobacco Institute to give credibility to its argument that smoking was a “disability” [43, pg.1].

**Table 2:** ADA training materials and publications by John Fox for the Tobacco Institute, 1991.

Type	Title	Publisher	Date
Report	Countdown to Compliance: What to do Now [44]	Pettit & Martin	April 1991
Summary	Executive Summary: The Americans with Disabilities Act as Applied to the Fire Community [45]	Pettit & Martin	April 1991
Report	Americans with Disabilities Act of 1991: A Comprehensive Analysis of Title I [46]	Pettit & Martin	May 1991
Briefing	Title III: The Americans with Disabilities Act [47]	Pettit & Martin	May 1991
Report	Americans with Disabilities Act Of 1990: Selected Parallel References to the ADA, Final Regulations and Interpretative Analysis [48]	Pettit & Martin	Aug 1991
Report	Affirmative Action Briefing: Americans with Disabilities Act of 1990 [49]	NELI	Oct 1991

In 1991, Fox participated in at least 17 seminars for the Tobacco Institute (Table 3). The seminars targeted business owners, managers, human resource staff and legal counsel [50] and provided opportunities to disseminate Fox’s materials (Table 2) [40,51,52]. Fox also worked with Walter Woodson (Tobacco Institute) and Lance Pressl (Philip Morris USA) to present on the ADA at the National Conference of State Legislatures in 1991 [53,54].

**Table 3:** ADA seminars for the Tobacco Institute involving John Fox, 1990–1992.

Seminar title	Date	Location	Host
Affirmative Action Briefing [39]	Oct 15–16, 1990	San Francisco, CA	NELI
Affirmative Action Briefing [39]	Oct 18–19, 1990	Seattle, WA	NELI
Affirmative Action Briefing [39]	Oct 25–26, 1990	San Antonio, TX	NELI
Affirmative Action Briefing [39]	Nov 1–2, 1990	Washington, D.C.	NELI
Affirmative Action Briefing [39]	Nov 5–6, 1990	Chicago, IL	NELI
Labor (re ADA and Performance Appraisals) [39]	Dec 4, 1990	San Francisco, CA	Pettit & Martin
Labor (re ADA and Performance Appraisals) [39]	Dec 6, 1990	San Jose, CA	Pettit & Martin
OFCCP and ADA [39]	Jan 25, 1991	Seattle, WA	AGC
Employment Law Briefing [39]	Mar 9–16, 1991	Vail, CO	NELI
Executive’s Role in Labor Relations [39]	April 18, 1991	Davis, CA	UC Davis
ADA–Countdown to Compliance [39]	April 19, 1991	Chicago, IL	NELI
ADA–Countdown to Compliance [39]	April 22, 1991	Washington, D.C.	NELI
The ADA as Applied to the Fire Community [45]	April 28, 1991	Rhode Island	Rhode Island Firefighters Meeting
ADA–Countdown to Compliance [39]	April 29, 1991	San Francisco, CA	NELI
Re ADA [39]	June 3, 1991	San Francisco, CA	Western Independent Bankers
Safety & Health Expo re. ADA [39]	June 5, 1991	Santa Clara, CA	America West
Beyond Workers’ Compensation–Healthy Workers Healthy Workplaces (re ADA) [39]	June 28, 1991	Vail, CO	National Conference of State Legislatures
The New Disabilities Law Countdown to Compliance: What to do Now [39]	Aug 13, 1991	San Francisco, CA	Pettit & Martin
The New Disabilities Law Countdown to Compliance: What to do Now [39]	Aug 14, 1991	San Jose, CA	Pettit & Martin
Affirmative Action Briefing [38]	Oct 10–11, 1991	Seattle, WA	NELI
Affirmative Action Briefing [38]	Oct 17–18, 1991	San Francisco, CA	NELI
Affirmative Action Briefing [38]	Oct 24–25, 1991	San Antonio, TX	NELI
Affirmative Action Briefing [38]	Oct 31-Nov 1, 1991	Chicago, IL	NELI
Affirmative Action Briefing [38]	Nov 7–8, 1991	Washington, D.C.	NELI
ADA Program [38]	April 6, 1992	San Francisco, CA	NELI
ADA Program [38]	April 10, 1992	Chicago, IL	NELI
ADA Program [38]	April 13, 1992	Washington, D.C.	NELI
Affirmative Action Briefing [55]	Oct 8–10, 1992	Seattle, WA	NELI
Affirmative Action Briefing [55]	Oct 15–17, 1992	San Francisco, CA	NELI
Affirmative Action Briefing [55]	Oct 22–24, 1992	Santa Fe, NM	NELI
Affirmative Action Briefing [55]	Oct 29–31, 1992	Chicago, IL	NELI
Affirmative Action Briefing [55]	Nov 5–7, 1992	Washington, D.C.	NELI

In January–September 1991, Fox and the Tobacco Institute used similar tactics to target fire departments. The Tobacco Institute considered this to be a priority issue because, at the time, fire departments were exempted from some state “smoker rights” laws on the basis that firefighters needed to be in good health, and therefore nonsmokers, to adequately perform their job [10]. Peter Sparber, a former Tobacco Institute employee who worked against proposals for fire-safe cigarettes, had good connections with fire departments [56]. In March 1991, Sparber told the Tobacco Institute how the ADA “may provide a relatively safe opportunity for us to reverse these [hiring] policies. Several fire service groups have begun to worry about the provisions of the [ADA] Act” [57]. Fox responded by writing a legal summary, “The Americans with Disabilities Act as Applied to the Fire Community” [45] and an article for the August 1991 issue of *Fire Engineering* [58].

#### Continuation of the Tobacco Institute’s ADA public relations program, 1992

In a 1992 plan, the Tobacco Institute discussed continuing its ADA public relations program: “although some of the tactics are already in action because of the natural flow of the issue, we need to continue the effort to guide the debate on the third-prong definition (‘perceived disability’) to encompass smokers” [25, pg.1]. The tactics included the media tours, seminars, and written materials Fox prepared in 1991.The Tobacco Institute was keen on maintaining focus on smoking as a perceived rather than actual disability, since, in the words of the Tobacco Institute, they would otherwise have to “pay [the] price of [an] ‘addiction’ label” [25, pg. 4].

The 1992 plan described two strategies. Strategy I was a continuation of the 1991 work, focusing on Fox’s employment law seminars and dissemination of his written materials [25]. Fox was to be paid $100,000 for legal seminars and writing projects, and another $100,000 for media tours and briefings. In 1992, Fox was involved in at least 8 seminars for the Tobacco Institute, all similar to those in 1990 and 1991 (Table 3) [55,59]. Although activity of the ADA public affairs program slowed in 1992, Fox reported to the Tobacco Institute that:

The Americans with Disabilities Act continues to deter corporate restrictions on the hire of smokers. A client employing over 70,000… has just reversed… its previous unpublished restriction on the hire of smokers… No publicity will occur and applicants will be none the wiser… the ADA is the most potent legal weapon I have yet identified to attack restrictive corporate smoking policies [60].

Strategy II was to “assist other interested organizations in pursuing court interpretations of the ADA” [25, pg. 5], focusing on work with disability rights groups [25]. The idea was to use these groups, often involved in ADA litigation, to identify and influence precedent-setting cases to win rulings that would help to push smokers under ADA coverage. The Tobacco Institute budgeted $30,000 in 1992 for the law firm Wunder, Ryan, Cannon & Thelen to liaise with disability rights groups [25]. Other Tobacco Institute reports suggest that disability or civil rights groups were used to promote the link between disability and smoking as part of the Tobacco Institute’s ADA public relations program [61], or to exert pressure on Congress to weaken the Armstrong Amendment [21].

Several civil rights groups, or individuals affiliated with them, were a part of the ADA Legislative Team (the civil rights activists who initially drafted the ADA bill in 1988) [29], or were involved in lobbying or ADA litigation after the ADA was signed into law. Some of these groups accepted money from the Tobacco Institute and other tobacco interests (Table 4).

**Table 4:** Groups involved in ADA legislative drafting, lobbying or litigation and accepting money from tobacco companies between 1988 and 1992.

Group name	Individuals affiliated with group & working on ADA	Amount USD, sponsor	Year	Details
The Dole Foundation [62]	Senator Robert Dole (R-KS) Paul Hearne, President of the Dole Foundation, Advisor in the ADA Legislative Team [29]	$54,000, multiple tobacco companies	1979–1994	Campaign contributions. Dole was Republican Minority leader when ADA was drafted. He founded the Dole Foundation in 1988.[9]
		Up to $6500/yr, Tobacco Institute	1988–1994	The Tobacco Institute supported the Dole Foundation to use Dole’s influence in the Senate.[63]
American Civil Liberties Union (ACLU) [64]	Chai Feldblum, Legislative Counsel to ACLU, Legal Coordinator in the ADA Legislative Team [29]	Over $1 million, Philip Morris USA	1987–1993	Some of the money was used to fund work on smoker’s rights including ADA employment protections for smokers,[65] and ADA litigation for people with obesity or AIDS so that smokers could be protected in a similar way by analogy.[9,66] Funding was kept quiet.[65]
Disability Rights Education and Defense Fund (DREDF)	Pat Wright, ADA Strategy and Team Coordinator; Arlene Mayerson, ADA Legal Coordinator; Mary Lou Breslin, Advisor in the ADA Legislative Team; Marilyn Golden, ADA Grass Roots Coordinator [29]	$3000, Tobacco Institute	1991	Purpose of funding was to sponsor the DREDF ADA Gala Celebration.[67,68] In DREDF promotional materials, the Tobacco Institute is not listed as a sponsor.[69]
Leadership Conference on Civil Rights (LCCR) [70]	Ralph Neas, Executive Director of LCCR and Advisor on ADA Legislative Team [29]	Unknown amount, Tobacco Institute	1989	LCCR was on the ADA Lobbying Team.
Paralyzed Veterans of America (PVA) [71]	Fred Cowell, ADA lobbying leader [29]	$10,000, Tobacco Institute	1989	Purpose of funding was to oppose smoking restrictions.

It is unclear how these funding relationships impacted the groups’ ADA-related activities, or whether individuals affiliated with these groups were directly or knowingly funded by tobacco companies. The exception was Senator Robert Dole (R-KS), the Republican Minority leader, whose influence in the Senate was valuable for the Tobacco Institute. In later years, the Tobacco Institute became reluctant to fund the Dole Foundation [72], because of Dole’s plans to retire from the Senate:

I think that Dole will leave the Senate at the end of the year whether he wins or loses the Presidency. In any event, he will be around for the rest of the year and still have influence on what happens, or doesn’t happen, in the Senate. Maybe we should do it once more and then let it go at that [63, pg. 1].

## Discussion

Congress was poised to exclude smoking from ADA coverage, but the Tobacco Institute, together with pressure from lawyer Forscey, Congressman Bliley and civil rights groups allied with the industry, altered language in the ADA “definition” and “construction” sections so that smokers might be covered by the ADA. Rewording was subtle and may have appeared irrelevant to tobacco control, but was intended to provide a legal basis to deter employers from hiring only non-smokers.

This “Trojan Horse” approach–where subtle legal language that appears irrelevant to tobacco or relatively innocuous may in fact have substantial policy impacts–has been employed by tobacco companies in other contexts, such as regulatory reform in the European Union [2] and “smoker’s rights” laws in the USA, framed as legislation to protect people from “lifestyle discrimination” [3]. Examples of similar tactics, in the context of regulations more closely intertwined with tobacco control, include tobacco industry involvement in the shaping of legal language in e-cigarette regulation [73] and tort law.[2,74]. The claim that smoking is a “disability” was a reach for the Tobacco Institute, because tobacco companies were still denying that nicotine was addictive. Nevertheless, the tobacco industry pursued the ADA as an opportunity to obtain legal protection for smokers by framing smoking as a perceived rather than actual disability. The ADA provides another strong example of why public health advocates and policymakers should be aware of the tobacco industry’s Trojan Horse tactics; not only in the context of tobacco legislation, but also laws seemingly unrelated to smoking such as civil rights legislation.

The ADA is ambiguous on whether smoking is a “disability,” and this ambiguity is a partly result of Tobacco Institute activities. Legal analyses and court cases indicate that the argument the Tobacco Institute tried to popularize–that smoking is a disability under the ADA–has little or no legal merit [3,6,15]. One court held that: “common sense compels the conclusion that smoking, whether denominated as 'nicotine addiction' or not, is not a 'disability' within the meaning of the ADA” [15]. Consistent with this ruling, Tobacco Institute lawyers had predicted that this argument would fail in courts [41]. Instead of pursuing ADA litigation, the Tobacco Institute mounted an ADA public relations program to convince employers that smokers were covered by the ADA, regardless of whether this argument was legally accurate. This inconsistency between legal advice and the resultant public message has been observed in other contexts, for example tobacco industry arguments regarding trademark law and international treaties [75].

Although the impacts of the Tobacco Institute’s ADA public relations program are difficult to quantify, Fox conducted at least 32 seminars across the country, reached over 100 fire departments with his article in *Fire Engineering*, and–according to Fox–convinced a company with over 70,000 employees to reverse a no-hire policy against smokers, with minimal publicity. These efforts were part of a broader tobacco industry scheme in the late 1980’s and early 1990’s to maintain the social acceptability of smoking in the workplace and to use legal strategies, often based on “smoker’s rights” arguments, to frame employer restrictions on smoking as unreasonable or unfair [7,8,76]. The legal arguments and solutions proposed in these strategies were often misleading for employers. For example, there is no “right to smoke” [77], and the tobacco industry’s “ventilation solution” to secondhand smoke did not protect the health of employees [78]. The argument that smokers are “disabled” under the ADA is yet another example of a false tobacco industry argument, intended to mislead employers into curtailing their own tobacco control efforts. Employers should remain alert to these tactics, and should not be deterred from encouraging a tobacco-free workforce on the basis that doing so would constitute a violation of the ADA.

Even the tobacco industry’s more attenuated arguments, such as the claim that smokers are “disabled,” could have important and broad-reaching impacts on policy and practice because of the subtle, skillful way they are implanted. The seminars conducted by Fox, for example, intended to create the idea that not hiring smokers would violate the ADA, but this argument was made in a subtle, indirect way, with Fox’s seminars advertised as having a broader focus. There was no mention of the tobacco industry’s involvement in these seminars or Fox’s ADA work for the industry in the available documents related to these seminars. This indirect approach has two effects. First, the tobacco industry’s role in shaping ideas is concealed since the issue is not directly presented as tobacco-related, thus making the idea appear more credible, especially if advanced by a qualified expert presented as independent of the tobacco industry (in this case, Fox). Second, presenting an idea in a subtle, indirect fashion (as opposed to pushing it to an audience harder or more directly) reduces the audience’s motivation to reject the idea (reactance), and therefore the likelihood they will question or critically engage with it [79]. Public health advocates and policymakers should remain alert to this tactic by remaining critical of broader ideas and arguments that may have broader policy implications in tobacco control, also if they are subtle or not obviously related to tobacco industry activity.

### Limitations

The main source of information for this paper was the Truth Tobacco Industry Documents Library, which is an incomplete collection based on documents produced through litigation against tobacco companies. Some document dates had to be estimated based on key events or dates on attachments to the documents, and matched with events in the Congressional records. One document was withheld as confidential attorney-client privilege, so the document content could not be viewed. Information was incomplete on the purpose and outcome of relationships between civil rights groups and the Tobacco Institute, and the impact of the ADA public affairs program on employer policies.

### Conclusion

The ADA’s ambiguous language on whether smoking is a disability was a result of pressure from the Tobacco Institute, its lawyers, and civil rights groups allied with the tobacco industry during legislative drafting of the ADA in 1989–1990. Language that would have clearly excluded smoking from ADA coverage was omitted or weakened. In 1991–1992, the Tobacco Institute used a public relations program to convince employers that smokers were protected by the ADA. These activities were part of a broader effort to prevent employers from taking active roles in tobacco control, and exemplary of more subtle, far-fetched tobacco industry tactics to influence policy and policy implementation while hiding the industry’s role in these activities. Employers and policy makers need to be aware that tobacco use is not a protected by the ADA and should not be misled by tobacco industry efforts to insinuate otherwise.
